# Signaling Pathways in Bone Development and Their Related Skeletal Dysplasia

**DOI:** 10.3390/ijms22094321

**Published:** 2021-04-21

**Authors:** Alessandra Guasto, Valérie Cormier-Daire

**Affiliations:** 1 Imagine Institute, Université de Paris, Clinical Genetics, INSERM UMR 1163, Necker Enfants Malades Hospital, 75015 Paris, France; alessandra.guasto@institutimagine.org; 2 Centre de Référence Pour Les Maladies Osseuses Constitutionnelles, Service de Génétique Clinique, AP-HP, Hôpital Necker-Enfants Malades, 75015 Paris, France

**Keywords:** bone development, signaling pathways, skeletal dysplasia

## Abstract

Bone development is a tightly regulated process. Several integrated signaling pathways including HH, PTHrP, WNT, NOTCH, TGF-β, BMP, FGF and the transcription factors SOX9, RUNX2 and OSX are essential for proper skeletal development. Misregulation of these signaling pathways can cause a large spectrum of congenital conditions categorized as skeletal dysplasia. Since the signaling pathways involved in skeletal dysplasia interact at multiple levels and have a different role depending on the time of action (early or late in chondrogenesis and osteoblastogenesis), it is still difficult to precisely explain the physiopathological mechanisms of skeletal disorders. However, in recent years, significant progress has been made in elucidating the mechanisms of these signaling pathways and genotype–phenotype correlations have helped to elucidate their role in skeletogenesis. Here, we review the principal signaling pathways involved in bone development and their associated skeletal dysplasia.

## 1. Introduction

Skeletal development in mammals occurs through two distinct mechanisms: intramembranous and endochondral ossification. In the intramembranous process, osteoblast cells differentiate directly from mesenchymal cells and form flat bones like cranial vault, parts of the jaw and lateral clavicles. At the end of the bone formation period, osteoblasts die by apoptosis or become embedded in the matrix as osteocytes, which then eventually undergo apoptosis [1]. In the endochondral process, which forms the rest of the skeleton, the mesenchymal cells condensate and differentiate in chondrocytes that deposit a cartilaginous model. Then, chondrocytes proliferate, forming the growth plate and secreting a cartilage-specific extracellular matrix. Afterwards, chondrocytes in the center of the cartilage arrest to divide and increase their volume, becoming hypertrophic chondrocytes [2,3]. The hypertrophic chondrocytes have the capacity to mineralize their extracellular matrix and can die by apoptosis or differentiate into osteoblasts. With the invasion of the cartilage model by blood vessels, stem cells of different lineages give rise to the bone depositing osteoblasts and the bone resorbing osteoclasts forming the primary ossification center. After formation of a secondary ossification center within the cartilaginous epiphyses during the early postnatal stage, the definitive growth plate can be identified by well-demarcated zones of cells representing the maturation steps of chondrocytes (resting, proliferative, prehypertrophic and hypertrophic) and remains, in humans, until puberty.

Both ossification processes are strictly regulated by several signaling pathways grouped by their response to the following signaling ligand families: Hedgehog (HH), Parathyroid Hormone-related Protein (PTHrP), Wingless and int-1 (WNT), NOTCH, Transforming Growth Factor-beta (TGF-β), Bone Morphogenic Protein (BMP) and Fibroblast Growth Factor (FGF). These pathways converge on specific transcription factors including SRY-related HMG-box 9 (SOX9), Runt-related transcription factor 2 (RUNX2) and Osterix (OSX). Dysregulation of these signaling pathways cause a large spectrum of skeletal diseases. Skeletal dysplasia (SD), also named osteochondrodysplasia, is a group of rare genetic disorders mainly characterized by cartilage and bone growth anomalies. Today, thanks to next-generation DNA sequencing, 461 different SD have been recognized and classified in 42 groups depending on their clinical, radiographic and/or molecular bases [4]. Here, we review the most important signaling pathways involved in bone development and the SD associated with signaling impairment. All SD mentioned in this review are listed in Table 1.

## 2. Hedgehog Signaling

**Figure 1:**
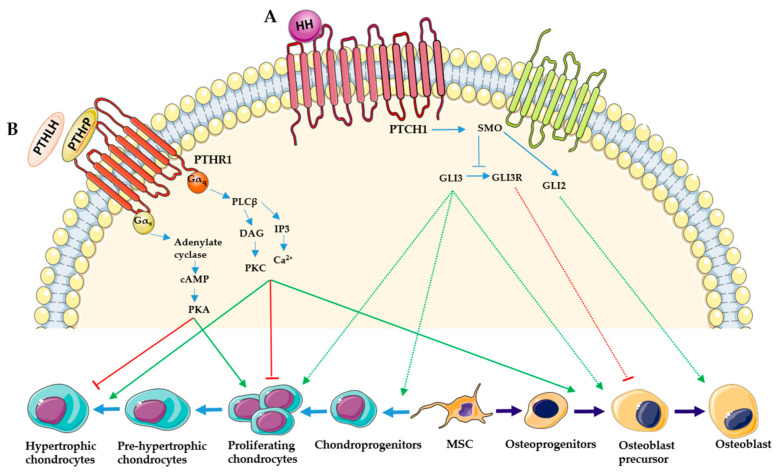
HH and PTHrP signaling pathways in chondrocyte and osteoblast differentiation (**A**) HH ligands interact with PTCH 1 receptor causing the activation of the SMO protein and in consequence, the activation of GLI transcriptor factors. HH signaling positively regulates the first steps of chondrogenesis from mesenchymal stem cells (MSC) and chondrocyte proliferation. It induces the specification of osteoblast precursors and the maturation in mature osteoblasts. The activation of the GLI3 repressor (GLI3R) inhibits osteoblast precursor proliferation. (**B**) PTHrP or PTHLH bind to PTHR1 activating Gαs and Gαq proteins. Through Gαs, PTHrP activates adenylate cyclase and the production of cAMP, which in turn activates PKA proteins inducing chondrocyte proliferation and inhibiting their hypertrophic differentiation. However, through Gαq, PTHrP activates PLCβ and the production of DAG and IP3, leading to the activation of PKC and to an increase of intracellular Ca^2+^, respectively. PLCβ signaling inhibits chondrocyte proliferation and induces their hypertrophic differentiation and osteoblast differentiation.

Hedgehog (HH) signaling is involved in many developmental processes, such as growth, patterning and morphogenesis of many tissues. In mammals, three HH ligands are produced: Sonic (SHH), Indian (IHH), and Desert Hedgehog (DHH). HH ligands interact with the 12-pass transmembrane Patched receptors (PTCH 1 and 2) causing the activation of the smoothened (SMO) protein, a 7- pass transmembrane protein with intrinsic intracellular activity. Its translocation to the base of primary cilium activates Gli transcriptor factors (GLI 1, 2 and 3) [5,6,7]. In vertebrates, SHH and IHH are the principal ligands involved in skeletal development. SHH is involved in the first stages of mesenchymal condensation. It promotes the epithelial-mesenchymal transition of sclerotome, the skeletogenic mesenchyme from which the intervertebral discs, vertebrae and ribs are derived [8,9]. SHH is also expressed in the developing face and regulates the outgrowth and the differentiation of the cranial-neural crest cell-derived skeletal structures [10,11]. Moreover, SHH have a critical role in digit patterning in the appendicular skeleton [12]. On the other hand, IHH is implicated in skeleton formation and in particular in endochondral ossification [5,6]. IHH is strongly expressed in the growth plate by pre- and early hypertrophic chondrocytes. It regulates chondrocyte hypertrophy in an indirect manner through a negative feedback loop with parathyroid hormone-related peptide (PTHrP), and directly promotes chondrocyte proliferation and osteoblast specification (Figure 1A) [13,14,15].

In the first case, IHH upregulates the expression of PTHrP, which maintains growth plate chondrocytes in a proliferative state and inhibits their hypertrophic maturation [16]. Indeed, the maintenance of proliferation promoted by PTHrP inhibits IHH production, since it is expressed by prehypertrophic chondrocytes and not by proliferative chondrocytes [15,17]. In the second case, IHH promotes chondrocyte proliferation in a direct manner by inhibiting the GLI3 activity and promoting the transition of round chondrocyte into proliferating chondrocytes [14,18].

HH is also involved in osteoblast development during ossification. In endochondral ossification, IHH produced by pre-hypertrophic and hypertrophic chondrocytes acts on osteoblast progenitors to induce the specification in Runx2-positive osteoblast precursors in the perichondrium and the primary spongiosa [19,20]. In addition to osteoblast differentiation in the perichondrium, HH signaling is also fundamental for trabecular bone formation [21].

As demonstrated by all these studies, HH signaling is critical for bone development and its misregulation results in several human skeletal diseases associated with a wide spectrum of limb and facial skeletal anomalies. In particular, as mentioned before, HH ligands diffuse across the developing limb bud and produce a concentration gradient that acts as a morphogen to specify digit development. HH signaling activating mutations, like those inactivating GLI3 repressor, cause polydactyly in Greig cephalopolysyndactyly syndrome, Pallister-Hall syndrome and Preaxial polydactyly type 4 [22,23,24,25,26], while missense heterozygous mutations in *IHH* cause brachydactyly type A1 [24,25,27]. Mutations in *IHH* have also been associated with Syndactyly with craniosynostosis [28], Syndactyly Lueken type [29] and acrocapitofemoral dysplasia [30]. This last disorder is caused by missense mutations causing an increased chondrocyte differentiation by diminishing IHH signaling in the growth plate. The phenotype is characterized by short stature with short limbs and highlights the importance of IHH in coordinating chondrocyte proliferation and differentiation. On the other hand, *SHH* mutations cause congenital hand deformities and severe craniofacial and neurological syndromes like Werner syndrome, Acheiropodia [31], different forms of polydactyly and syndactyly [32], and Laurin-Sandrow syndrome [33].

## 3. Parathyroid Hormone-Related Protein

PTHrP is a paracrine factor expressed by cartilage and bone. In particular, PTHrP is expressed by periarticular resting cells of the perichondrium and at a lower level by proliferating chondrocytes [19,34]. PTHrP binds to a specific receptor (PTHR1), which is G-protein coupled and is composed of seven transmembrane domains [35]. PTHR1 is expressed at low levels by proliferating chondrocytes and at a higher level in prehypertrophic chondrocytes [17,19]. Upon ligand binding, receptor activation results in stimulation of the heteromeric (αβγ) guanine nucleotide binding proteins (Gα_s_ and Gα_q_). Through Gα_s_, PTHrP receptor activates adenylate cyclase (AC) and the production of 3’, 5’-adenosine monophosphate (cAMP), which in turn activates protein kinase A (PKA) [27,36]. On the other hand, stimulation of Gα_q_ activates phospholipase Cβ (PLCβ) and the formation of diacylglycerol (DAG) and 1,4,5-inositol triphosphate (IP3). DAG activates in turn proteinase kinase C (PKC), while the production of IP3 leads to an increase of intracellular Ca^2+^ [37,38].

These two signaling pathways have opposite actions on chondrocyte differentiation (Figure 1B). PTHrP uses AC/cAMP/PKA pathway to keep chondrocyte proliferating and to promote the activation of the transcriptor factor SOX9, that restrains chondrocyte differentiation [39,40,41]. In addition, PKA inhibits the expression of the myocyte enhancer factor 2 (MEF2) and of RUNX2, two key transcription factors promoting chondrocyte hypertrophy via the recruitment of the class II histone deacetylase HDAC4 in the nucleus [42]. In contrast, PLC/IP3 signaling via the PTH/PTHrP receptor appears to restrain proliferation of chondrocytes and to stimulate their progression toward hypertrophic differentiation [43]. PTHrP is also produced by early osteoblast precursors and actively synthetizing osteoblasts. It has been showed that periosteum-derived PTHrP induced osteoblast activity and bone formation [44,45,46].

The critical role of PTHrP receptor in bone development is highlighted by the discovery of two severe chondrodysplasias: Blomstrand lethal chondrodysplasia and metaphyseal chondrodysplasia Jansen type. Inactivating recessive *PTHR1* mutations in Blomstrand dysplasia cause prenatal lethality, shortened limbs, advanced bone mineralization and increased bone density due to reduction of proliferating and resting chondrocytes within the growth plate [47,48]. Metaphyseal chondrodysplasia Jansen type, meanwhile, is caused by activating mutations that lead to ligand-independent activation of PTHR1 and of the cAMP pathway. This causes a phenotype characterized by severe abnormalities of the growth plate, short-limbed dwarfism, micrognatia and hypercalcemia [49]. Interestingly, a homozygous truncating mutation in *PTHR1* has been identified in Eiken dysplasia, a disorder with opposite phenotype compared to Blomstrand dysplasia. Patients with Eiken dysplasia show severely delayed skeletal maturation, as well as mild growth retardation [50]. These findings demonstrate that distinct recessive mutations in the same gene can generate contrasting phenotypes. Another connection between human genetic bone disorder and PTHrP signaling is shown in brachydactyly type E, caused by loss-of-function mutations in parathyroid hormone-like hormone (PTHLH), a PTHR1 ligand, and characterized by bone shortening of hands and feet [51]. A more severe brachydactyly phenotype is also found in acrodysostosis, in association with facial dysostosis, nasal hypoplasia and developmental delay. This disease is caused by mutations in *PRKAR1A*, which is the type 1 regulatory subunit of PKA or mutations in phosphodiesterase 4 (*PDE4D*), both causing an impaired PKA activation [52,53]. Furthermore, inactivating mutations in the maternal allele of *GNAS* gene, coding for the binding protein Gα_s_, cause Pseudohypoparathyroidism type IA, also known as Albright hereditary osteodystrophy (AHO) [54,55]. This disease is characterized by short stature, subcutaneous ossifications and brachydactyly, and is associated with PTH resistance. If the same mutations are inherited from the father, the AHO phenotype occurs without hormone resistance [56]. It has been proposed that the defective Gα_s_-mediated hypertrophy inhibition could result in promotion of the Gα_q_-mediated chondrocytes’ hypertrophy, leading to premature growth plate closure and favoring short stature.

On the other hand, McCune-Albright syndrome or isolated fibrous dysplasia (FD) of bone is caused by gain-of-function mutations in distinct sites of the *GNAS* gene compared to AHO [57,58]. These mutations cause overproduction of cAMP in a ligand-independent manner in osteogenic cells. This leads to the accelerated production of bone marrow stromal cells, while inhibiting the differentiation of these progenitors into mature osteoblasts [59]. These immature cells lead to under-mineralized bone with disrupted micro-architecture. Finally, deletions of the chromosomal region 2q37, which include the *HDAC4* gene, cause brachydactyly–mental retardation syndrome characterized by brachydactyly type E, craniofacial abnormalities and intellectual disabilities [60].

## 4. Wingless and Int-1

**Figure 2:**
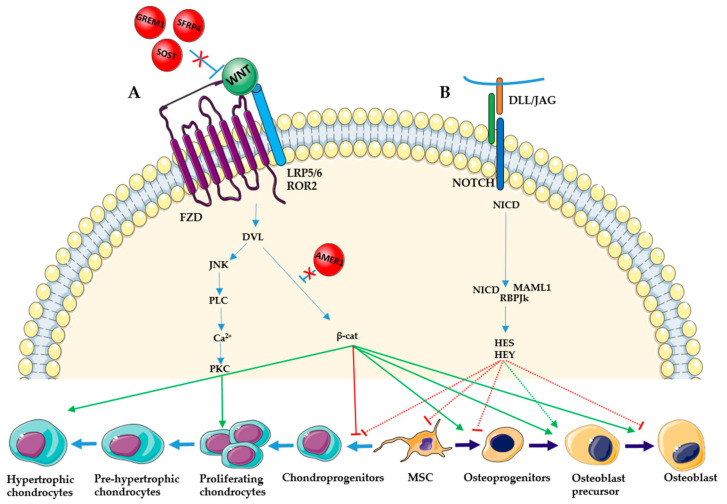
WNT and NOTCH signaling pathways in chondrocyte and osteoblast differentiation. (**A**) WNT ligands bind to the FZD receptors with or without specific co-receptors like LRP5, LRP6 and ROR2. In the canonical pathway, WNT binding activates the translocation of β-catenin in the nucleus. This inhibits the first step of chondrogenesis but induces the chondrocyte hypertrophic differentiation and osteoblast differentiation and maturation. The non-canonical pathways, through the activation of DVL, JNK and PLC proteins, activate PKC and induce chondrocyte proliferation. WNT signaling can be negatively regulated by extracellular inhibitors, such as SOST, GREM1 and SFRP4, and by intracellular inhibitors, like AMER1. (**B**) In the NOTCH pathways, DLL or JAG ligands bind to NOTCH receptor and cause consequent proteolytic cleavages resulting in the release of NICD which translocate in the nucleus and interact with MAML1 and RBPJk proteins. NOTCH signaling inhibits chondrogenesis, and the first and the last stages of osteoblast differentiation, but promotes the differentiation of osteoprogenitors in osteoblast precursors.

WNT is a complex regulator of skeletal development. Until now, three different WNT pathways have been identified: one canonical pathway, the WNT/β-catenin, and two non-canonical pathways, the WNT/planar cell polarity (PCP) and the WNT/Ca^2+^. All these signaling pathways are activated by the binding of extracellular WNT ligands to one of the seven-pass transmembrane Frizzled (FZD) receptors. Depending on the pathway, the activation occurs with or without specific co-receptors like lipoprotein-related protein (LRP) or receptor tyrosine kinase-like orphan receptors (ROR) [61,62]. In the canonical pathway, WNT ligands bind the receptor complex formed by LRP5/6 and FZD. This activation causes the translocation of β-catenin in the nucleus where it activates WNT target genes controlling proliferation and differentiation. When WNT is absent, β-catenin is bound in a complex composed by glycogen synthase kinase 3β (GSK3β), axin and adenomatosis polyposis coli (APC) and degraded by proteasome. In the non-canonical PCP pathway, Jun kinase (JNK) is activated via disheveled (DVL) and the RAC and Rho small GTPases. Finally, in the non-canonical WNT/Ca^2+^ pathway, WNT binds to FZD receptors and ROR causing the intracellular Ca^2+^ release and the activation of some calcium-sensitive enzymes. More downstream, this event activates the nuclear factor of activated T cells (NF-AT), which translocates to the nucleus and induces specific gene expressions. WNT signaling pathways play a central role in bone development regulating chondrocyte and osteoblast differentiation (Figure 2A).

WNTs secreted by the ectoderm act via a β-catenin-dependent pathway. It blocks the cartilage formation in limb bud mesenchymal cells, by inhibiting SOX9 expression through a repressive chromatin mark (H3K27me3) and DNA methylation over the *SOX9* promoter [63]. The inactivation of β-catenin causes an increase of SOX9 expression, leading to a decreased chondrocyte proliferation and a delayed hypertrophic maturation. In addition, the PCP pathway plays a unique role in inducing chondrocyte column formation in the growth plate [64,65]. WNT can also promote chondrocyte hypertrophy by inhibiting PTHrP activity [43]. Some members of the WNT family, such as WNT5A and WNT5B, regulate the transition of chondrocytes to hypertrophy in the growth plate. It has been shown, that the absence of WNT5A delays chondrocyte hypertrophy and that a proper level of WNT5A/B signaling is required for the normal transition of chondrocytes to hypertrophy [66,67]. Some WNT proteins inhibit chondrogenesis from mesenchymal stem cells (MSC), favoring osteoblastogenesis. WNT has a primary role in osteoblast differentiation, since the activation of the β-catenin pathway is essential to allow the differentiation of mesenchymal progenitors in mature osteoblasts. Several studies confirm that WNT activation and inhibition correspond to the increase and the decrease of the bone mass, respectively [68,69,70,71].

WNT ligands consist of 19 different cysteine-reach secreted glycoproteins. Several of them have been associated with SD. For example, loss-of-function mutations in *WNT1*, involved in the canonical pathway, are responsible for osteogenesis imperfecta (OI) type XV and for early onset osteoporosis [72]. Loss-of-function of *WNT3*, playing an essential role in axis formation and limb growth, causes tetra-melia syndrome type 1, consisting on the absence of all four limbs [73]. The *WNT5A* gene, involved in the WNT/PCP non canonical pathway, is linked to the autosomal dominant form of Robinow syndrome (RS) type 1 [74]. Mutations in *WNT6* have been identified in acro-pectoro-vertebral dysplasia (F-syndrome) and mutations in *WNT7A* gene, involved in limb development and contributing to anteroposterior patterning, have been identified Al-Awadi-Raas-Rothscild syndrome and Fuhrmann syndrome. Finally, mutations in the *WNT10B* gene, a key regulator of osteogenesis, playing an important role in the development of hands and feet, cause split-hand/foot malformation type 6 [75,76,77,78]. Mutations have been also identified in WNT receptors and co-receptors: with FZD2, being associated with omodysplasia type 2; ROR2 with autosomal dominant brachydactyly type 1, or autosomal recessive RS type 1; and LRP4 with sclerostosis type 2 and Cenani-Lenz syndrome. Interestingly, mutations in the co-receptor LRP5 result in opposite bone mass phenotype. Loss-of-function mutations in LRP5 lead to decreased binding of WNT ligands and decreased WNT signaling activity causing osteoporosis. In contrast, other specific mutations in LRP5 affecting the binding of Dickkopf-1, an inhibitor of WNT signaling, result in gain-of-function of LRP5 and in osteosclerosis [79,80,81,82,83,84,85,86,87,88]. Extracellular WNT inhibitors and activators play also a significant role in bone homeostasis and development. This is demonstrated by the identification of mutations in the WNT inhibitor SOST disrupting the ability of SOST to link with LRP co-receptors. This generates an increased WNT signaling and an increased bone mass like in sclerosteosis type 1, Van Buchem diseases and craniodiaphyseal dysplasia. Furthermore, Cenani-Lenz-like non-syndromic bilateral oligosyndactyly and Pyle disease have been associated with mutations in the WNT inhibitors GREM1 and SFRP4 respectively. Loss-of-function mutations in *AMER1* (or *WTX*), an intracellular inhibitor of WNT are responsible for X-linked osteopathia striata with cranial sclerosis [89]. Finally, Keipert syndrome and tetra-amelia syndrome are caused by loss-of-function mutations in the WNT activators GPC6 and RSPO2, respectively [78].

The discovery and the understanding of all these diseases showed how WNT signaling is essential to regulate bone density in humans, since its hyperactivation causes skeletal diseases characterized by high-bone mass phenotype, while its inhibition causes decreased bone formation. Furthermore, it explains why WNT is used as therapeutic target to improve bone mass in patients affected by skeletal diseases such as osteoporosis or osteogenesis imperfecta.

## 5. Notch

Similar to WNT/β-catenin signaling, Notch signaling also suppresses chondrogenesis through four NOTCH receptors (NOTCH1-4) and at least five ligands (two members of the Jagged family: Jag1 and Jag2 and three members of the Delta-like family: Dll1, Dll3, and Dll4) [90]. In the canonical Notch pathway, the ligands bind to the NOTCH receptors present on the adjacent cell surface. This triggers one first proteolytic cleavage generating a Notch Extracellular Truncation (NEXT) and a consequent proteolytic cleavage mediated by the γ-secretase complex, composed by Presenilin 1 and 2. This cleavage results in the release of the Notch Intracellular Domain (NICD) in the cytoplasm [91,92]. NICD translocates into the nucleus where it forms a complex with the transcription factor CSL (C promoter-binding factor 1, Suppressor of Hirless and Lag1), also known as RBPJk, and the transcriptional coactivator Mastermind-like 1 (MAML1). This ternary complex activates the expression of transcription factors of the families Hairy Enhancer of Split (HES 1, 4, 5, 6 and 7) and Hes related with YRPW motif (HEY 1, 2 and L) [93]. Multiple NOTCH ligands and receptors are expressed in the prechondrogenic mesenchyme and inhibit both mesenchymal condensation and subsequent chondrocyte differentiation (Figure 2B) [94,95]. This occurs when Hey and Hes transcription factors are activated and bind to *SOX9* enhancer and suppress the *SOX9* gene expression, which is essential for chondrocyte differentiation [96,97].

Notch signaling is also critical for axial skeleton segmentation during somitogenesis [98,99]. Defective somitogenesis, due to abnormal Notch signaling, cause Spondylocostal dysostosis and vertebral segmentation defect characterized by vertebrae and ribs anomalies with consequent trunk dwarfism [100,101]. These pathologies have been associated with mutations in different components of Notch signaling, such as DLL3, HES7, Mesoderm Posterior (MESP) 2, a Notch target gene, and Lunatic Fringe (LFNG), a member of the Fringe family, involved in the post-translational modifications of NOTCH receptors [102,103,104,105].

In parallel, Notch signaling acts upstream of RUNX and regulates the osteochondro-progenitor cell proliferation and lineage specification [106]. Depending on the osteoblast differentiation stage, Notch signaling can promote or inhibit the process [107]. In the early stage of osteoblastogenesis, the Hey transcriptional factors, downstream of RBPjK, inhibit the Runx2 expression and, in consequence, prevent the differentiation of osteochondroprogenitor cells into perichondral cells [108,109]. Meanwhile, during the intermediate stage, the expression of NCID promotes proliferation and the differentiation of perichondral cells into preosteoblasts. Finally, in the late stage, Notch inhibits the preosteoblast differentiation in mature osteoblasts [110]. To resume, Notch signaling inhibits the early and late stage of osteoblastogenesis but promotes the intermediate stage. Consistent with this description, gain-of-function mutations in *NOTCH2*, the major receptor involved in the chondrocyte and osteoblast differentiation, are responsible for Hadju–Cheney syndrome, a disorder characterized by focal bone lysis of distal phalanges, progressive bone loss and fractures [111,112]. Loss-of-function mutations in *NOTCH1*, *RBPJ* and *DLL4* are responsible for Adams Oliver syndrome, a congenital condition characterized by aplasia cutis congenita of the scalp and transverse limb defects [113,114].

Since the Notch pathway can have different roles depending on the cell differentiation stage, it could be very difficult to target this pathway for therapies.

## 6. Transforming Growth Factor-Beta and Bone Morphogenetic Protein

**Figure 3:**
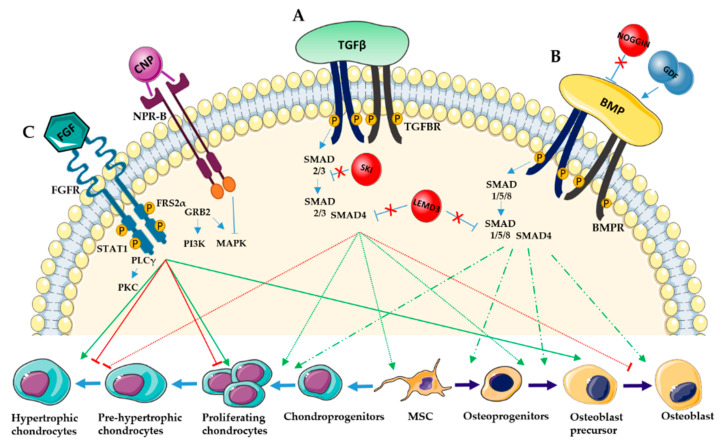
TGFβ, BMP and FGF signaling pathways in chondrocyte and osteoblast differentiation. (**A**) TGF-β ligands bind to TGFBR type 1 and 2 causing the phosphorylation of the receptors and of SMAD 2 and 3 proteins which interact with SMAD4. This complex accumulates in the nucleus and induces chondrogenesis and osteoblastogenesis but inhibits the last step of chondrocyte and osteoblast maturation. SMAD activity can be blocked by intracellular inhibitors like SKI and LEMD3. (**B**) BMP ligands bind to BMPR type 1 and 2 causing the phosphorylation of the receptors and of SMAD 1, 5 and 8 proteins which interact with SMAD4. This complex accumulates in the nucleus and induces chondrocyte proliferation and all the steps of osteoblast differentiation. GDF ligands and the NOGGIN antagonist also bind BMPR. (**C**) FGF ligands bind FGFR causing receptor transphosphorylation, the phosphorylation of the adaptor protein FRS2α and the activation of STAT1, PLCγ and GRB2. GRB2 in turn activates PI3K and MAPK proteins. Through these pathways, FGF promotes chondrocyte proliferation and differentiation in the first step of the development and it promotes osteoblast proliferation and differentiation. In contrast, in the later stages of development, it inhibits chondrocyte proliferation and differentiation. MAPK signaling, downstream of FGFR, can be negatively regulated by the CNP-NPR pathway.

The TGF-β superfamily is a group of more than 40 members including 3 TGF-β proteins (TGF-β1, TGF-β2 and TGF-β3), and 14 BMPs, Activins and Growth and Differentiation Factors (GDFs) [115]. It is involved in two principal pathways, TGF-β and BMP signaling. In both cases, the binding of the TGF-β superfamily ligands to a heteromeric serine/threonine receptor ligand complex formed by two type I (TGFBRI, BMPRI or ALK) and two type II receptors (TGFBR2 or BMPR2) causes the autophosphorylation of type II receptors. This in turn phosphorylate type I receptors and the regulatory Smad transcription factors (R-SMADS). These R-SMADS form a complex with the coregulatory Smad (Co-SMAD), SMAD4, which accumulates in the nucleus and regulates gene transcription [116,117]. TGF-β signaling mostly involves SMAD 2 and 3 proteins, whereas BMP signaling is principally mediated by SMAD 1, 5, and 8. As an alternative to the SMAD-mediated canonical pathways, TGF-β and BMP also activate p38 MAPK family cascade through non-canonical pathways. TGF-β, BMPs and their receptors play a relevant role in all stages of bone development (Figure 3A,B).

This has been demonstrated by studying several conditional knockout mouse models for TGF-β, its receptors and SMAD proteins. All these models are characterized by an impairment of endochondral and intramembranous bone formation [118].

During chondrogenesis, BMP and TGF-β signaling are required for the expression and maintenance of SOX9 and, in consequence, for the formation of chondrogenic mesenchymal condensations. TGF-β, BMPs and their receptors are also expressed in proliferating chondrocytes and hypertrophic chondrocytes and regulate chondrocyte proliferation and differentiation. In particular, TGF-β promotes the condensation and differentiation of mesenchymal cells, induces chondrocyte proliferation, but inhibits terminal chondrocyte differentiation [119,120]. On the contrary, BMP signaling is not essential for the mesenchymal condensation, but is indispensable for mesenchymal cell differentiation and for chondrocyte proliferation and maturation [121,122].

In osteoblastogenesis, TGF-β promotes expansion and proliferation of osteoprogenitors, early differentiation and commitment to the osteoblastic lineage through the MAPKs and SMAD 2/3 pathways. Conversely, it prevents the terminal osteoblast differentiation [123]. Activating mutations in the genes coding for the ligand TGF-β2 and 3, for the TGF-β receptors (TGFBR1 or TGFBR2), and for the intracellular SMAD2 and 3 have all been associated with the Loeys-Dietz syndrome, an autosomal dominant condition characterized by skeletal deformities and bone overgrowth [124]. Mutations in the gene coding for the ligand TGF-β1 and causing the hyperactivation of the TGF-β signaling are responsible for the Camurati-Engelmann disease, characterized by increased bone density [125,126].

Focusing on BMP signaling, genetic studies have shown that several BMPs, such as BMP-2, 4, 5, 6 and 7 have strong osteogenic capacity [127]. In particular, BMP-2, with the coordinated help of BMP-4, controls the transition from RUNX2- to OSX- positive osteoblasts. On the other hand, BMP-7 promotes the expression of osteoblast differentiation markers as ALP activity and enhances calcium mineralization. Its absence in postnatal bones can be compensated by the others BMPs. Instead, BMP-3 inhibits skeletal progenitor cell differentiation to mature osteoblasts and regulates adult bone mass [118].

In humans, activating mutations in one of the Type I receptors, the Activin receptor IA/Activin-Like kinase 2 (ACVR1/ALK2), cause Fibrodysplasia ossificans progressive (FOP), a rare disease characterized by heterotopic endochondral ossification, through an increased BMP Smad-dependent and non-canonical p38 MAPK signaling activity [128]. Mutations in BMPR1 (or ALK6), which is specifically expressed during chondrogenesis and osteoblastic differentiation, cause brachydactyly type A2 and acromesomelic dysplasia. Both diseases have been also associated with loss-of-function mutations in *GDF5* [129]. Patients with these diseases have normal axial skeletons but missing or fused distal elements in hands and feet. These discoveries highlighted the importance of BMP signaling in distal limb patterning. Disruptions of the BMP pathway also causes abnormal axial skeletal patterning. GDF6, a close relative to GDF5, is associated with Klippel-Feil syndrome causing bone fusions [130]. Gain-of-function mutations in *SMAD4*, the only common SMAD for both TGF-β and BMP signaling, results in Myhre syndrome, characterized by short stature, facial dysmorphism, stiff joints and brachydactyly [131].

The TGF-β/BMP signaling is also negatively regulated by several mechanisms: intracellular antagonists, like SMAD 6 and 7; the inner nuclear membrane protein LEMD3 and the SKI protein influencing the SMAD signaling; extracellular ligand antagonists, like NOGGIN and BMPER; intracellular ubiquitin ligases and transcriptional repressors. Over-expression of NOGGIN causes osteopenia, fractures and multiple synostoses syndrome, consisting of facial dysmorphism, craniosynostosis and multiple joint fusion [132]. Heterozygous missense mutations in the antagonist NOGGIN, leading to an increased inhibitory activity, also result in brachydactyly type B [133]. Mutations in *BMPER* cause diaphanospondylodysostosis characterized by craniofacial anomalies and vertebral segmentation [134]. Moreover, loss-of-function mutations in the *LEMD3* gene are associated with osteopoikilosis with and without melorheostosis, characterized by multiple round foci of increased bone density and hyperostosis respectively [135]. Finally, loss-of-function mutations in the SKI protein, which inhibits SMAD2/3 activation, are responsible for Shprintzen Goldberg Syndrome, characterized by marfanoid habitus and craniosynostosis [136].

As mentioned in this paragraph, numerous human SD are related to TGF-β/BMP signaling pathways confirming their essential role in bone development. In particular, it seems that increased TGF-β signaling can result in either increased bone formation or craniosynostosis. Indeed, the clinical consequences depend on tissue-specificity expression of the mutant allele and on the interaction with other signaling pathways. On the other hand, impaired BMP signaling mostly causes distal and axial skeletal defects, demonstrating a prominent role of BMP in chondrocyte differentiation and establishment of joint boundaries.

## 7. Fibroblast Growth Factor

Fibroblast growth factors (FGFs) are a large family of 18 or more proteins which mostly bind to four cell surface tyrosine kinase FGF receptors (FGFR 1-4). This binding induces the dimerization of the receptor tyrosine kinase domains and their sequential trans-phosphorylation [137]. Consequently, it promotes the phosphorylation of the adaptor proteins FGFR substrate 2α (FRS2α) and the recruitment of other adaptor proteins like signal transducer and activation of transcription1 (STAT1), phospholipase γ (PLCγ) and the guanine nucleotide exchange factor GRB2. These proteins activate multiple signaling pathways, including JAK-STAT, proteinase kinase C (PKC), mitogen-activated protein kinase (MAPK) and phosphoinositide 3-kinase (PI3K), [138].

FGFs and their receptors are expressed during all stages of the bone development in a time- and space-dependent manner. For example, FGFR1 is expressed during mesenchymal condensation, in perichondrium and periosteum and in hypertrophic chondrocytes of the growth plate [139]. FGFR2 is initially expressed at high levels in condensed mesenchyme and then in perichondrium and periosteum [140]. FGFR3 is expressed in proliferative chondrocytes in the growth plate and, when it binds to its ligands FGF9 or FGF18, it promotes chondrocyte proliferation and the initial chondrocyte hypertrophy during the early stages of development [141,142]. On the contrary, it inhibits chondrocyte proliferation and hypertrophy in the later stages of development (Figure 3C) [143,144,145].

FGF signaling is fundamental also in intramembranous ossification and in the regulation of all steps of osteoblastogenesis. In particular, FGFR1 is expressed in calvaria mesenchyme and later in osteoblasts. During early development, it promotes osteoblast differentiation without affecting RUNX2 expression, while, in mature osteoblasts, it inhibits their mineralization activity [139]. In contrast, FGFR2, expressed in differentiating osteoblasts, promotes the proliferation of preosteoblasts and the anabolic function of mature osteoblasts mostly by up-regulating RUNX2 expression [146,147]. Finally, mice lacking *Fgfr3* showed an increase in osteoblast number but a decrease in osteoid mineralization [148].

Mutations in *FGFRs* cause human skeletal dysplasia with a variable degree of severity. The most common genetic skeletal dysplasia in humans is achondroplasia, a rhizomelic short limb dwarfism characterized by short stature, limited elbow extension and frontal bossing. It is caused by gain-of-function mutations in the *FGFR3* gene leading to an overactivation of the FGF signaling [149,150,151]. Since in the growth plate FGFR3 is predominantly expressed in proliferating and prehypertrophic chondrocytes, these mutations cause a reduced chondrocyte proliferation and an impaired hypertrophic differentiation [152,153,154]. Related chondrodysplasia syndromes include the milder form of dwarfism, hypochondroplasia, characterized by short stature and increased head circumference [155,156,157], the more severe and lethal forms, thanathophoric dysplasia type I and II, and the severe achondroplasia with developmental delay and acantosis nigricans (SADDAN) [158,159]. All these syndromes are caused by gain-of-function mutations in different domains of the FGFR3 protein and the phenotype severity is linked to the degree of FGFR3 activation [153]. On the contrary, inactivation of *FGFR3* cause skeletal overgrowth, campodactyly, tall stature and hearing loss (CATSHL) syndrome [160,161] and Lacrimoauriculodentodigital (LADD) syndrome. This last disorder is characterized by micrognatia and variable digit defects, and has been also associated with mutations in *FGFR2* [162]. The role of FGFR3 in intramembranous ossification is highlighted by the identification of *FGFR3* mutations in craniosynostosis disorders like Crouzon syndrome with acanthosis nigricans and Muenke syndrome [163,164]. Craniosynostosis, characterized by premature fusion of cranial sutures due to increased osteoblast proliferation or differentiation, or to increased mineralizing functions of osteoblasts, are also caused by mutations in *FGFR1* and *FGFR2*. Crouzon, Apert, Beare-Stevenson with cutis gyrata, Bent-bone dysplasia, Saethre-Choetzen, Jackson-Weiss and Pfeiffer syndromes are all caused by activating mutation in *FGFR2* gene and the consequent accelerated osteoblast maturation in the sutures [165,166,167]. Jackson-Weiss and Pfeiffer syndrome have also been associated with mutation in *FGFR1*, like the Hartsfield syndrome and the osteoglophonic dysplasia [168,169,170,171]. Finally, mutations in genes encoding FGF ligands have also been identified in SD, like FGF9 in multiple synostosis syndrome type 3, FGF10 in LADD and FGF23 in hypophosphatemic rickets [172,173,174]. In this last disorder, FGF23 overexpression causes defects in osteocytes and abnormal kidney function. This demonstrates that signaling defects can be tissue and organ nonautonomous [175].

During bone development, the FGF signaling can be modulated by C-type natriuretic peptide (CNP) action. When CNP binds to its NPR-B receptor, the MAPK signaling activated by FGFR3 is inhibited (Figure 3C). Similarly, the binding of FGF2 and FGF18 ligands to FGFR reduces CNP action [176,177]. CNP is expressed in proliferating and pre-hypertrophic chondrocytes and promotes chondrocyte proliferation and differentiation. Dominant negative and gain-of-function mutations in *NPR2*, the gene encoding NPR-B, are responsible for short and tall stature phenotype, such as in acromesomelic dysplasia type Maroteaux and tall stature with long halluces type NPR2, respectively [178,179]. These studies suggest that CNP signaling promotes chondrocytes proliferation during bone development and that it acts as a negative cross talk between FGF and MAPK. For this reason, recombinant analogues of CNP are used as promising therapy for achondroplasia. 

## 8. Transcription Factors 

### 8.1. SOX9

SOX9 is a transcription factor of the SRY family that regulates sex determination and developmental events like cartilage development. SOX9 is the earliest nuclear factor required for chondrogenesis. It is initially expressed in mesenchymal condensations of the early skeletal precursors. It is dispensable for the initial mesenchymal condensation, but crucial for the subsequent chondrocyte differentiation. In fact, in its absence, chondrogenesis is blocked. In the growth plate, SOX9 is expressed at highest levels in proliferating and prehypertrophic chondrocytes and less expressed in chondrocytes undergoing hypertrophy [2,180]. SOX9 directly activates chondrocyte differentiation markers like collagen type II. It also induces the expression of SOX5 and SOX6 working with SOX9 to activate the chondrocyte differentiation [181]. In addition, SOX9 expression sustains chondrocyte survival through PI3K-AKT pathway and maintains proliferation of columnar chondrocytes. It also prevents the entering of columnar chondrocytes in the prehypertrophic stage and promotes the subsequent hypertrophy [182]. SOX9 represses the initial chondrocyte maturation through the interaction with β-catenin and its consequential disruptions. This leads to the inhibition of the WNT pathway, a promoter of chondrocyte hypertrophy [183,184]. SOX9 can also directly interact and block the activity of RUNX2 (discussed below), one of the main transcription factors involved in chondrocyte maturation and osteoblastogenesis. Finally, SOX9 regulates the gene expression of specific markers for hypertrophic chondrocytes, like collagen type X and Vegfa [182,185,186,187]. SOX9 plays an important role in the developing axial skeleton and maintains the notochord and the demarcation of intervertebral disc compartments. Heterozygous loss-of-function mutations in *SOX9* gene have been associated with campomelic dysplasia, an autosomal dominant skeletal dysplasia characterized by hypoplastic scapulae, progressive disc degeneration, small chest, short long bones with bowing of the lower extremities, Pierre Robin sequence and sex reversal in males [188,189,190].

### 8.2. RUNX2

RUNX2 is a transcription factor belonging to the RUNX family composed by RUNX1, RUNX2 and RUNX3 [191]. RUNX2 is essential for both intramembranous ossification and endochondral ossification during skeletogenesis. It is involved principally in chondrocyte and osteoblast differentiation and in the regulation of extracellular matrix protein expression during these processes. It is initially expressed within the chondrogenic mesenchyme, subsequent to and dependent on SOX9 expression [192]. During endochondral bone development, RUNX2 is weakly expressed in proliferative chondrocytes and highly expressed in prehypertrophic chondrocytes until terminal hypertrophic chondrocytes and in perichondrium [193,194]. It regulates chondrocyte hypertrophy by driving the expression of collagen type X in hypertrophic chondrocytes, of matrix metallopeptidase 13 (MMP13) in terminal hypertrophic chondrocytes and also by interacting with BMP-regulated SMADs [195,196,197,198]. Moreover, RUNX2 controls chondrocyte maturation, enhancing FGF18 expression in the perichondrium [199]. In endochondral and intramembranous bones, RUNX2 is strongly detected in osteoblast progenitors, immature and early mature osteoblasts. It is required for osteoblast progenitor proliferation, their differentiation into osteoblasts, and for the proper function of the mature osteoblasts [200,201,202]. Haploinsufficiency of *RUNX2* causes cleidocranial dysplasia, characterized by short stature, delayed closure of fontanelles, prominent forehead, drooping shoulders and abnormal dental development. The distinctive radiological features are shortened or absent clavicles, delayed ossification of the skull bones, and delayed ossification of pelvic bones [203,204]. On the other hand, duplications of the *RUNX2* gene are associated with metaphyseal dysplasia with maxillary hypoplasia, characterized by metaphyseal flaring of long bones, enlargement of the medial halves of the clavicles, maxillary hypoplasia, and dystrophic teeth [205,206].

### 8.3. OSX

OSX, a transcription factor containing three C2H2-type zinc fingers, controls the genetic program of osteoblast differentiation and bone formation. Expression of OSX in osteoblast precursors induces the differentiation of these cells into mature and functional osteoblasts and, then, into osteocytes [207,208]. OSX can also be a negative regulator of chondrocyte differentiation. In fact, when OSX is expressed in RUNX2-positive cells, the expression of SOX9 is down-regulated and the cells exist as osteoblast precursors. Then RUNX2/OSX-positive cells differentiate into mature osteoblasts, in which SOX9 is not expressed. This means that without the expression of OSX, the RUNX2-positive precursors do not progress into the preosteoblast stage but, instead, they remain in the chondrogenic stage [209]. OSX also plays an important role in extracellular matrix calcification by activating MMP13 expression in hypertrophic chondrocytes [210]. A homozygous mutation in the *OSX* gene has been identified in a patient with a moderate form of osteogenesis imperfecta, presenting with bone fractures, mild bone deformities and delayed tooth eruption. This confirms the importance of a proper OSX expression for bone health [211].

## 9. Conclusions

In this review, we discussed the main signaling pathways involved in bone development and how mutations in their components have been associated with SD. It is important to highlight that even if the signaling pathways have been discussed independently, there is a complex cross-talk among them at multiple levels. This, in association with the evidence that the mutation consequences depend on the specificity of the mutations and on their temporal and spatial mode of action, makes more difficult the understanding of the physiopathological mechanisms of these diseases. Moreover, these signaling pathways can be secondarily affected by alterations in other cellular processes, such as extracellular matrix regulation or metabolic processing. Indeed, several skeletal dysplasia, that we decided to omit in this review, have been associated with mutations in these processes. Fortunately, in the last decade, the development of new technologies, like whole exome and genome sequencing has accelerated the identification of skeletal dysplasia-causing mutations. On the other hand, the development of CRISPR-Cas9 technology and of several mouse models is helping the deciphering of the physiopathological mechanisms. Advanced genetic testing is also helping the diagnosis of skeletal dysplasia. The diagnosis and management of these pathologies have long been based on clinical feature and skeletal imaging. Today, these key techniques are increasingly combined with the genetic testing in order to obtain a more accurate and early diagnosis of SD. It also aids in prognosis and in counselling families regarding genetic recurrence risk and preconceptional reproductive planning [212,213,214]. These continuous discoveries will help to expand the genotype–phenotype correlation of SD and to develop new therapeutic strategies. Nowadays, few treatments are available for SD, but several clinical trials are ongoing to validate new drugs targeting specifically these pathways in achondroplasia or FOP for example, and highlighting the importance of multidisciplinary cross talks (from bed to bench side) [215].

## Figures and Tables

**Table 1:** Signaling pathways involved in bone development and associated skeletal dysplasia.

Signaling Pathway/Transcription Factor	Associated Disease	Gene(s)	OMIM Number
HH	Werner syndrome	*SHH*	188,740
	Acheiropodia	*SHH*	200,500
	Preaxial polydactyly type 1 (PPD1)	*SHH*	174,400
	Preaxial polydactyly type 2 (PPD2)	*SHH*	174,500
	Syndactyly type 4 (I-V) Haas type	*SHH*	186,200
	Laurin-Sandrow syndrome	*SHH*	135,750
	Preaxial polydactyly type 4 (PPD4)	*GLI3*	174,700
	Pallister-Hall syndrome	*GLI3*	146,510
	Grieg cephalopolysyndactyly	*GLI3*	175,700
	Acrocapitofemoral dysplasia	*IHH*	607,778
	Syndactyly with craniosynostosis (Philadelphia type)	*IHH*	185,900
	Syndactyly Lueken type	*IHH*	
	Brachydactyly type A1	*IHH*	112,500
PTHrP	Blomstrand dysplasia	*PTHR1*	215,045
	Metaphyseal dysplasia, Jansen type	*PTHR1*	156,400
	Eiken dysplasia	*PTHR1*	600,002
	Acrodysostosis	*PDE4D* *PRKAR1A*	614,613 101,800
	Brachydactyly type E	*PTHLH*	613,382
**Signaling pathway/Transcription factor**	**Associated disease**	**Gene(s)**	**OMIM number**
PTHrP	Pseudohypoparathyroidism type IA (Albright hereditary osteodystrophy)	*GNAS*	103,580
	Fibrous dysplasia, polyostotic form (McCune–Albright)	*GNAS*	174,800
	Brachydactyly–mental retardation syndrome	*HDAC4*	600,430
WNT	Osteogenesis imperfecta, progressively deforming type (OI type 3)	*WNT1*	615,220
	Osteogenesis imperfecta, moderate form (OI type 4)	*WNT1*	615,220
	Tetra-amelia syndrome type 1	*WNT3*	273,395
	Robinow syndrome dominant type	*WNT5A*	180,700
	Acro-pectoro-vertebral dysplasia (F-syndrome)	*WNT6*	102,510
	Al-Awadi Raas-Rothschild limb-pelvis hypoplasiaaplasia	*WNT7A*	276,820
	Furhmann syndrome	*WNT7A*	228,930
	Split-hand-foot malformation, isolated form, type 6 (SHFM6)	*WNT10B*	225,300
	Robinow syndrome recessive type	*ROR2*	268,310
	Brachydactyly type B	*ROR2*	113,000
	Cenani-Lenz syndactyly	*LRP4*	212,780
	Osteoporosis-pseudoglioma syndrome	*LRP5*	259,770
	Osteosclerosis	*LRP5*	144,750
	Osteoporosis-AD form	*LRP5* *WNT1*	166,710 615,220
	Osteopetrosis type 1	*LRP5*	607,634
	Sclerosteosis	*SOST* *LRP4*	269,500 614,305
**Signaling pathway/Transcription factor**	**Associated disease**	**Gene(s)**	**OMIM number**
WNT	Endostheal hyperostosis, van Buchem type	*SOST*	239,100
	Craniodiaphyseal dysplasia	*SOST*	122,860
	Cenani-Lenz-like non-syndromic bilateral oligosyndatyly	*GREM1*	
	Pyle disease	*SFRP4*	265,900
	Osteopathia striata with cranial sclerosis (OSCS)	*AMER1*	300,373
	Tetra-amelia syndrome type 2	*RSPO2*	618,021
	Omodysplasia type 1	*GPC6*	258,315
NOTCH	Spondylocostal dysostosis	*DLL3* *MESP2* *HES7* *LFNG*	277,300 608,681 613,686 609,813
	Vertebral segmentation defect (congenital scoliosis) with variable penetrance	*MESP2* *HES7*	608,681 613,686
	Hajdu-Cheney syndrome	*NOTCH2*	102,500
	Adams-Oliver syndrome	*NOTCH1* *DLL4* *RBPJ*	616,028 616,589 614,814
BMP/TGFβ	Multiple synostoses syndrome	*NOG* *GDF5* *GDF6*	186,500 610,017 617,898
	Brachydactyly type B2	*NOG*	611,377
	Klippel-Feil syndrome	*GDF6*	118,100
	Acromesomelic dysplasia (Grebe dysplasia)	*GDF5* *BMPR1B*	200,700 609,441
	Acromesomelic dysplasia (Fibular hypoplasia and complex brachydactyly, Du Pan)	*GDF5* *BMPR1B*	228,900
**Signaling pathway/Transcription factor**	**Associated disease**	**Gene(s)**	**OMIM number**
BMP/TGFβ	Diaphanospondylodysostosis	*BMPER*	608,022
	Fibrodysplasia ossificans progressive (FOP)	*ACVR1*	135,100
	Diaphyseal dysplasia Camurati Engelmann	*TGFB1*	131,300
	Myhre syndrome	*SMAD4*	139,210
	Loeys-Dietz syndrome (types 1–6)	*TGFBR1* *TGFBR2* *SMAD3* *TGFB2* *TGFB3* *SMAD2*	609,192 610,168 613,795 614,816 615,582 601,366
	Brachydactyly type A2	*BMPR1B* *BMP2* *GDF5*	112,600 112,600 112,600
	Brachydactyly type C	*GDF5*	113,100
	Osteopoikilosis	*LEMD3*	166,700
	Melorheostosis with osteopoikilosis	*LEMD3*	166,700
	Shprintzen–Goldberg syndrome	*SKI*	182,212
FGF	Thanatophoric dysplasia type 1	*FGFR3*	187,600
	Thanatophoric dysplasia type 2	*FGFR3*	187,601
	SADDAN	FGFR3	616,482
	Achondroplasia	*FGFR3*	100,800
	Hypochondroplasia	*FGFR3*	146,000
	Camptodactyly, tall stature and hearing loss syndrome (CATSHL)	*FGFR3*	610,474
	Bent bone dysplasia	*FGFR2*	614,592
	Osteoglophonic dyplsasia	*FGFR1*	166,250
	Pfeiffer syndrome	*FGFR1* *FGFR2*	101,600 101,600
	Jackson-Weiss syndrome	*FGFR2* *FGFR1*	123,150 123,150
	Apert syndrome	*FGFR2*	101,200
	Craniosynostosis with cutis gyrata (Beare-Stevenson)	*FGFR2*	123,790
	Crouzon syndrome	*FGFR2*	123,500
**Signaling pathway/Transcription factor**	**Associated disease**	**Gene(s)**	**OMIM number**
FGF	Saethre–Chotzen syndrome	*FGFR2*	
	Crouzon-like with achantosis nigricans	*FGFR3*	612,247
	Craniosynostosis Muenke type	*FGFR3*	602,849
	Multiple synostosis syndrome type 3	*FGF9*	612,961
	Hartsfield syndrome	*FGFR1*	615,465
	Lacrimo-auriculo-dento-digital syndrome (LADD)	*FGFR2* *FGFR3* *FGF10*	149,730
	Hyperostosis–Hyperphosphatemia syndrome	*FGF23*	617,993
	Acromesomelic dysplasia type Maroteaux	*NPR2*	602,875
	Tall stature with long halluces, NPR2 type	*NPR2*	615,923
SOX9	Campomelic dysplasia	*SOX9*	114,290
RUNX2	Metaphyseal dysplasia with maxillary hypoplasia	*RUNX2*	156,510
	Cleidocranial dysplasia	*RUNX2*	119,600
SP7/Osterix	Osteogenesis imperfecta, moderate form (OI type 4)	*SP7*	613,849

## Data Availability

Not applicable.
